# Supplementary material for: Multiway Analysis of the Electrochemical Oxidation Pathway of a Lignin Using Chemometrics
Source: Molecules. 2025 Nov 5;30(21):4305. doi: 10.3390/molecules30214305 (PMC12608362; doi:10.3390/molecules30214305)
Supplement: Supplementary file 1 [file molecules-30-04305-s001.zip › molecules-3748715-supplementary.pdf]

Supporting Information

MULTIWAY ANALYSIS OF THE ELECTROCHEMICAL OXIDATION PATHWAY OF A LIGNIN USING CHEMOMETRICS

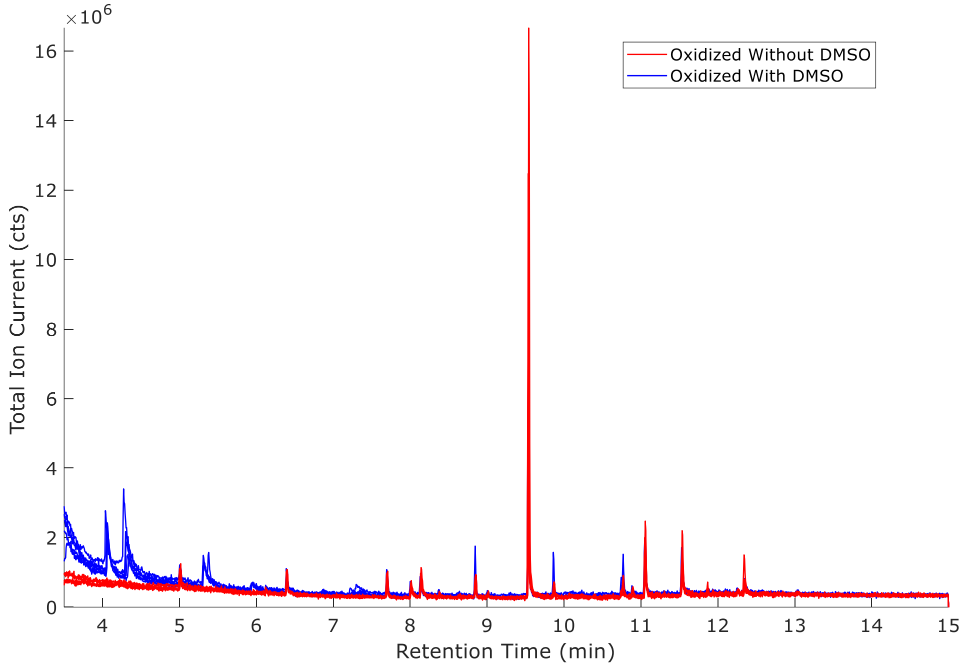

Figure S1. Total Ion Current (TICs) for $O_{0.8}$ oxidized (red, without DMSO) and $O^{D}_{0.8}$ oxidized samples (blue, with DMSO). Solvent: ACN + NaOH (1:3, v/v); voltage: 0.8 V vs. Hg/HgO; the two oxidized samples were measured with five replicates.

(a)

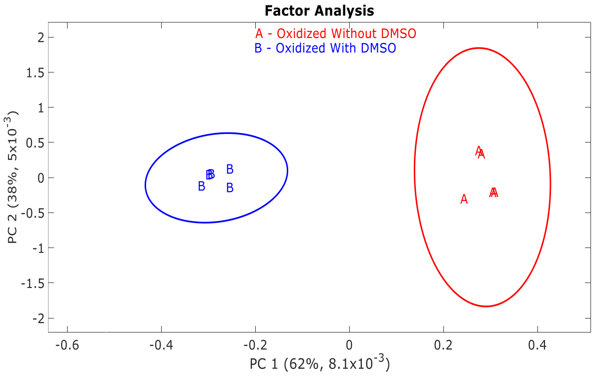

(b)

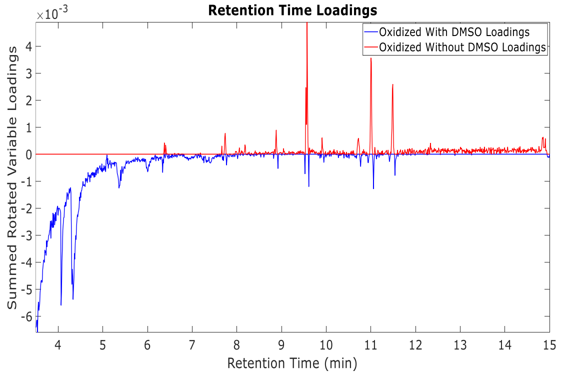

Figure S2. (a) Factor of the distributions of the five replicates for $O_{0.8}$ oxidized (red, no DMSO) and $O^{D}_{0.8}$ oxidized samples (blue, with DMSO) and **(b)** Retention time loadings.

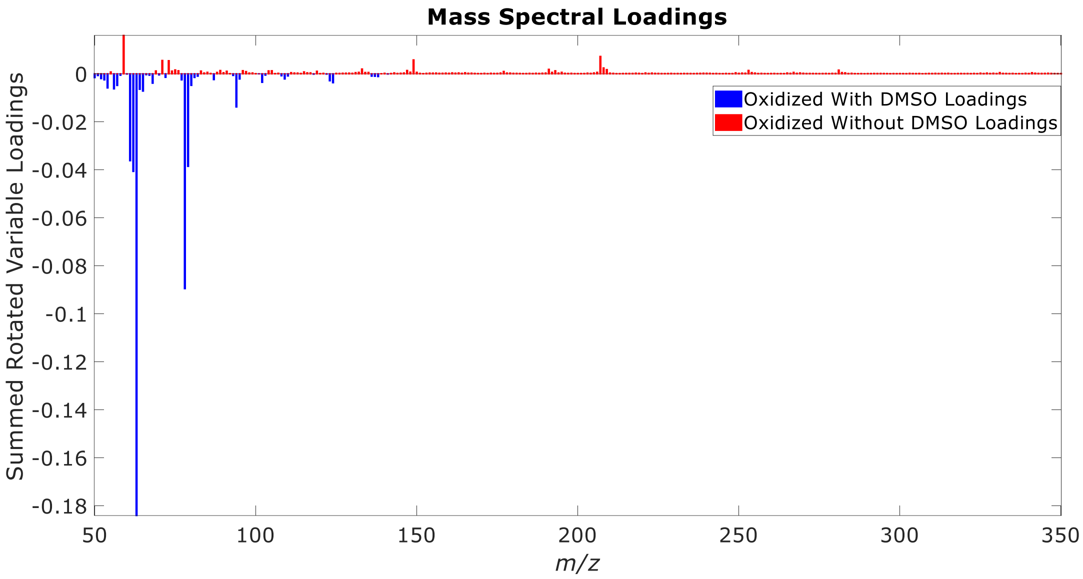

Figure S3. Mass spectral loadings of $O_{0.8}$ (red, without DMSO) and $O^{D}_{0.8}$ (blue, with DMSO) oxidized samples.

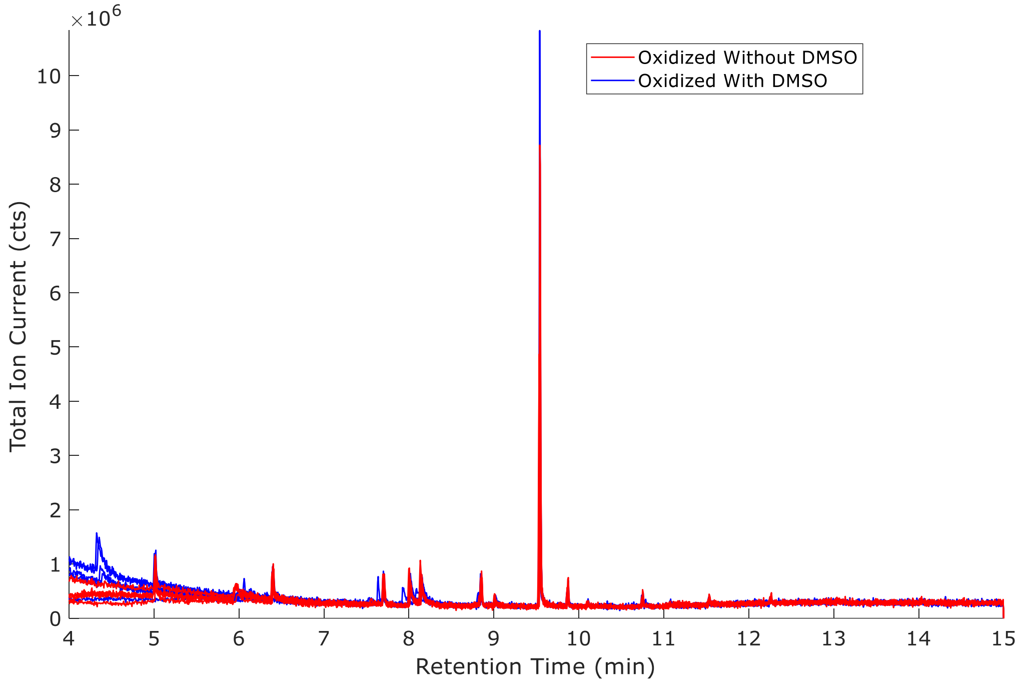

Figure S4. TICs for $O_{0.6}$ (red, without DMSO) and $O^{D}_{0.6}$ (blue, with DMSO) oxidized samples. Solvent: ACN + NaOH (1:3, v/v); voltage: 0.6 V vs. Hg/HgO; the two oxidized samples were measured with five replicates.

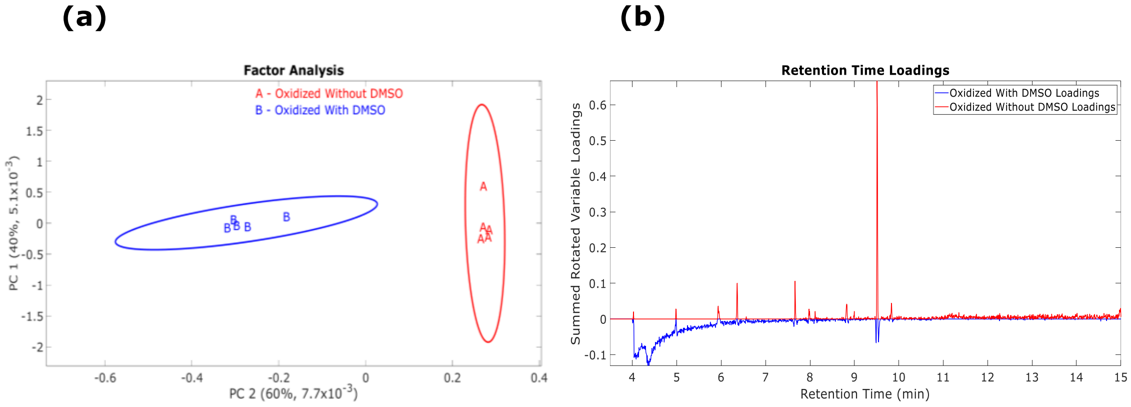

Figure S5. (a) Factor analysis to visualize the distributions of the five replicates for $O_{0.6}$ (red, without DMSO) and $O^{D}_{0.6}$ (blue, with DMSO) oxidized samples, and **(b)** Retention time loadings.

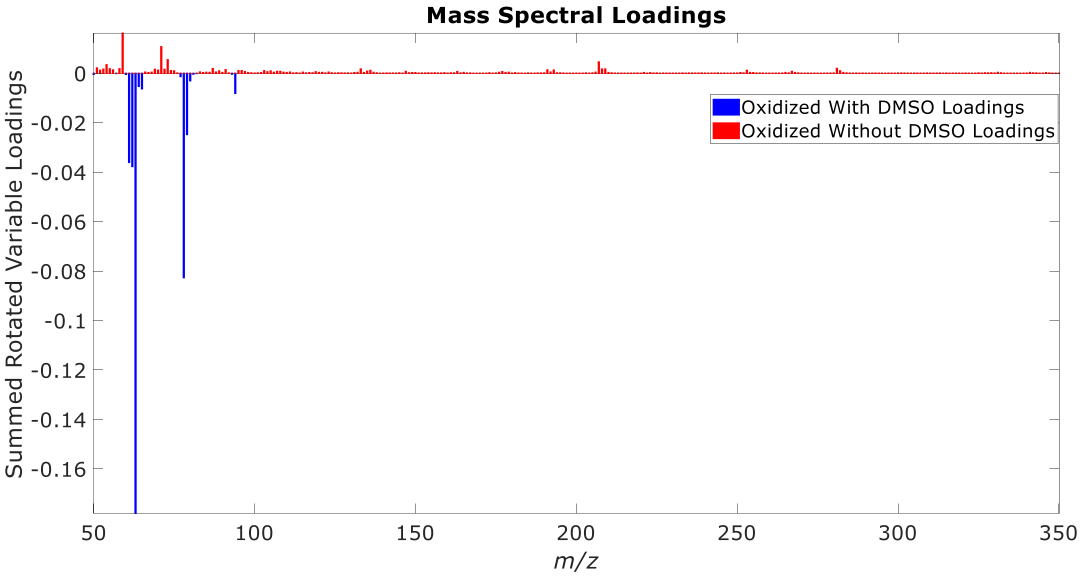

Figure S6. Mass spectral loadings of $O_{0.6}$ (red, without DMSO) and $O^{D}_{0.6}$ (blue, with DMSO) oxidized samples with five replicates.

Table S1. Oxidation Products for Sample $O_{0.8}$ (0.8 V vs. Hg/HgO, Without DMSO).

Retention Time (min)	QRM	Similarity	Compound Name	Structure
**8.14**	13.44	0.70	Propanoic acid, 2-methyl-, heptyl ester	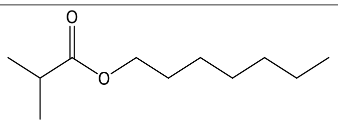
**9.54**	42.03	0.83	Furan, 2-butyltetrahydro-	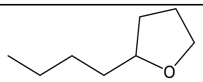
**10.76**	27	0.87	9-Octadecenoic acid (z)	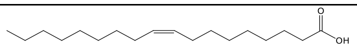
**11.53**	60	0.96	1,2-Benzenedicarboxylic acid, dibutyl ester	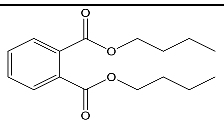

Table S2. Oxidation Products for Sample $O^{D}_{0.8}$ (0.8 V vs. Hg/HgO, With DMSO).

Retention Time (min)	QRM	Similarity	Compound Name	Structure
**5.38**	82.39	0.86	Cyclohexene,1-methyl-4-(1-methylethenyl)	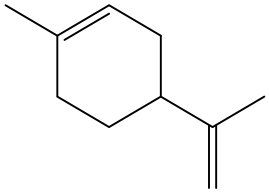
**8.14**	98.90	0.86	4,4-Dimethyl-3-oxa-5.alpha.-cholestan-2-one	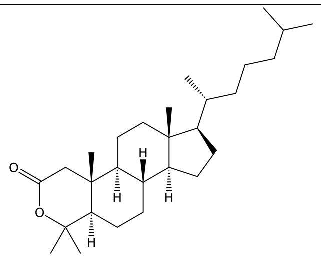
**9.01**	40.00	0.84	Phenol, 2,4-bis(1,1-dimethylethyl)-	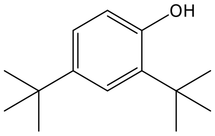
**9.54**	24.18	0.99	Furan, 2-butyltetrahydro-	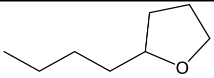
**11.06**	98.19	0.88	1,2-benzenedicarboxylic acid, bis(2-methylpropyl) ester	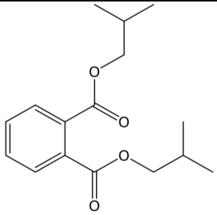
**11.35**	0.56	0.52	trans-2-Phenyl-1,3-dioxolane-4-methyl octadec-9,12,15-trienoate	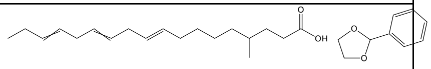

Table S3. Oxidation Products for Sample $O_{0.6}$ (0.6 V vs. Hg/HgO, Without DMSO).

**Retention Time (min)**	**QRM**	**Similarity**	**Compound Name**	**Structure**
**5.38**	82.39	0.86	Cyclohexene, 1-methyl-4-(1-methylethenyl)	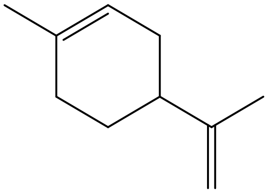
**8.02**	34.46	0.90	1,3-Butanediol, 2-methyl-	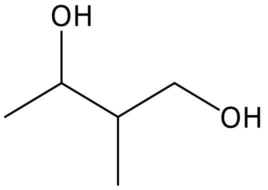
**8.14**	67.26	0.96	Butanoic acid, 3-hydroxy-2,2-dimethyl-, hexyl ester	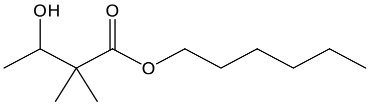
**9.01**	40.00	0.84	Phenol, 2,4-bis-(1,1-dimethylethyl)-	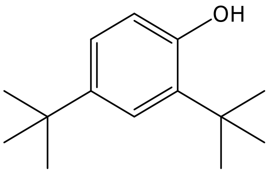
**9.54**	46.10	0.84	Furan, 2-butyltetrahydro-	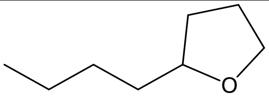

Table S4. Oxidation Products for Sample $O^{D}_{0.6}$ (0.6 V vs. Hg/HgO, With DMSO).

**Retention time (min)**	**QRM**	**Similarity**	**Compound Name**	**Structure**

**8.02**	9.52	0.93	Propanoic acid, 2-methyl- 2,2-dimethyl-1-(2-hydroxy-1-methylethyl)-, propyl ester	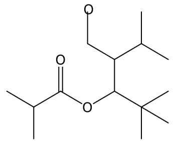
**8.09**	100	0.95	Butanoic acid, 3-hydroxy-2,2-dimethyl-, hexyl ester	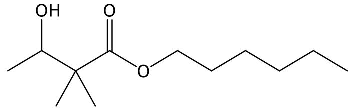
**9.01**	99.86	0.58	Phenol, 2,4-bis(1,1-dimethylethyl)-	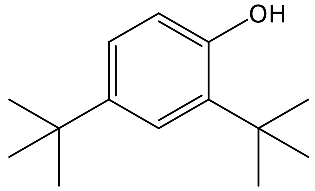
**9.54**	5.11	0.95	Furan, 2-butyltetrahydro-	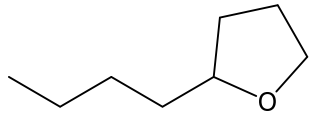
